# Supplementary figures and images for: Microbial Community Colonization Process Unveiled through eDNA-PFU Technology in Mesocosm Ecosystems
Source: Microorganisms. 2023 Oct 5;11(10):2498. doi: 10.3390/microorganisms11102498 (PMC10609261; doi:10.3390/microorganisms11102498)

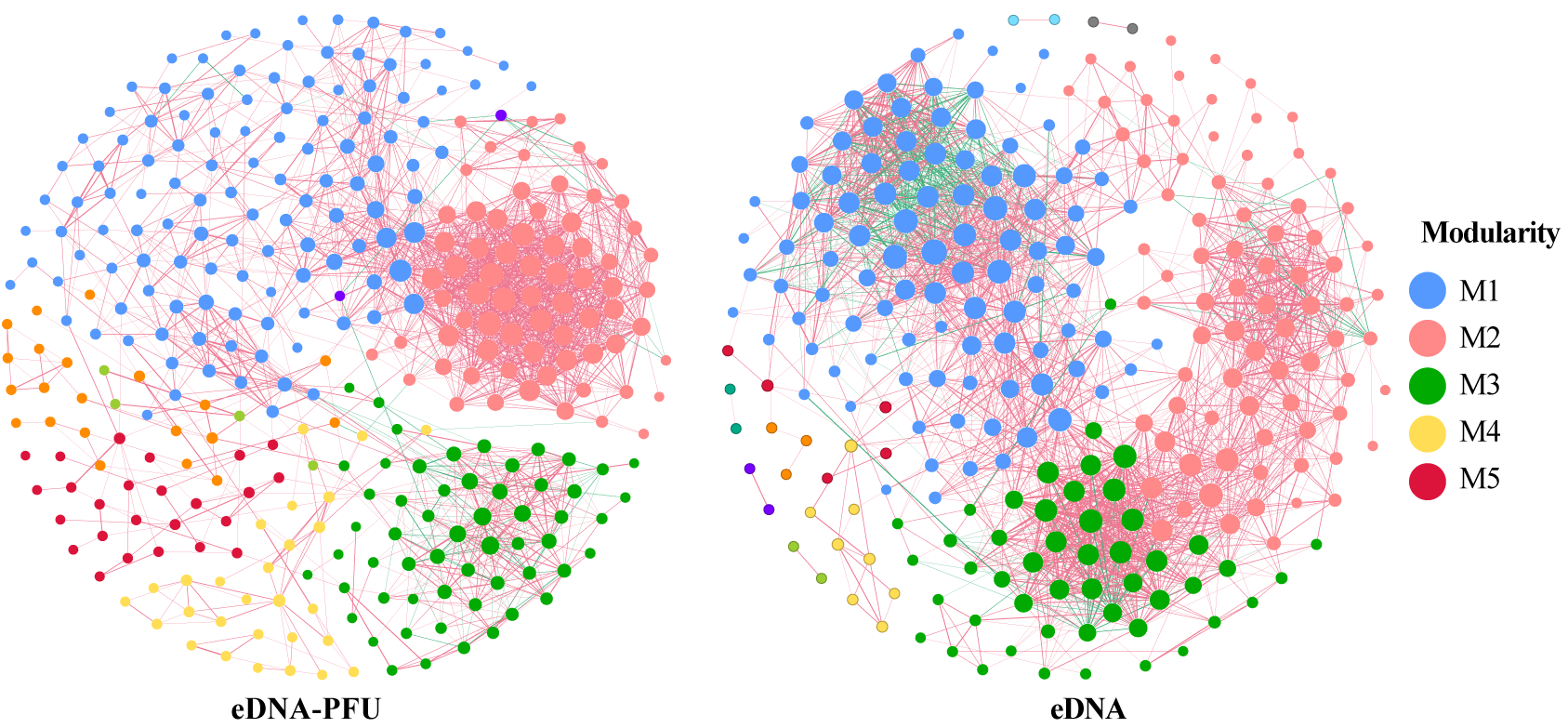

Supplement: Supplementary file 1 [file microorganisms-11-02498-s001.zip › FigureS1.tif]

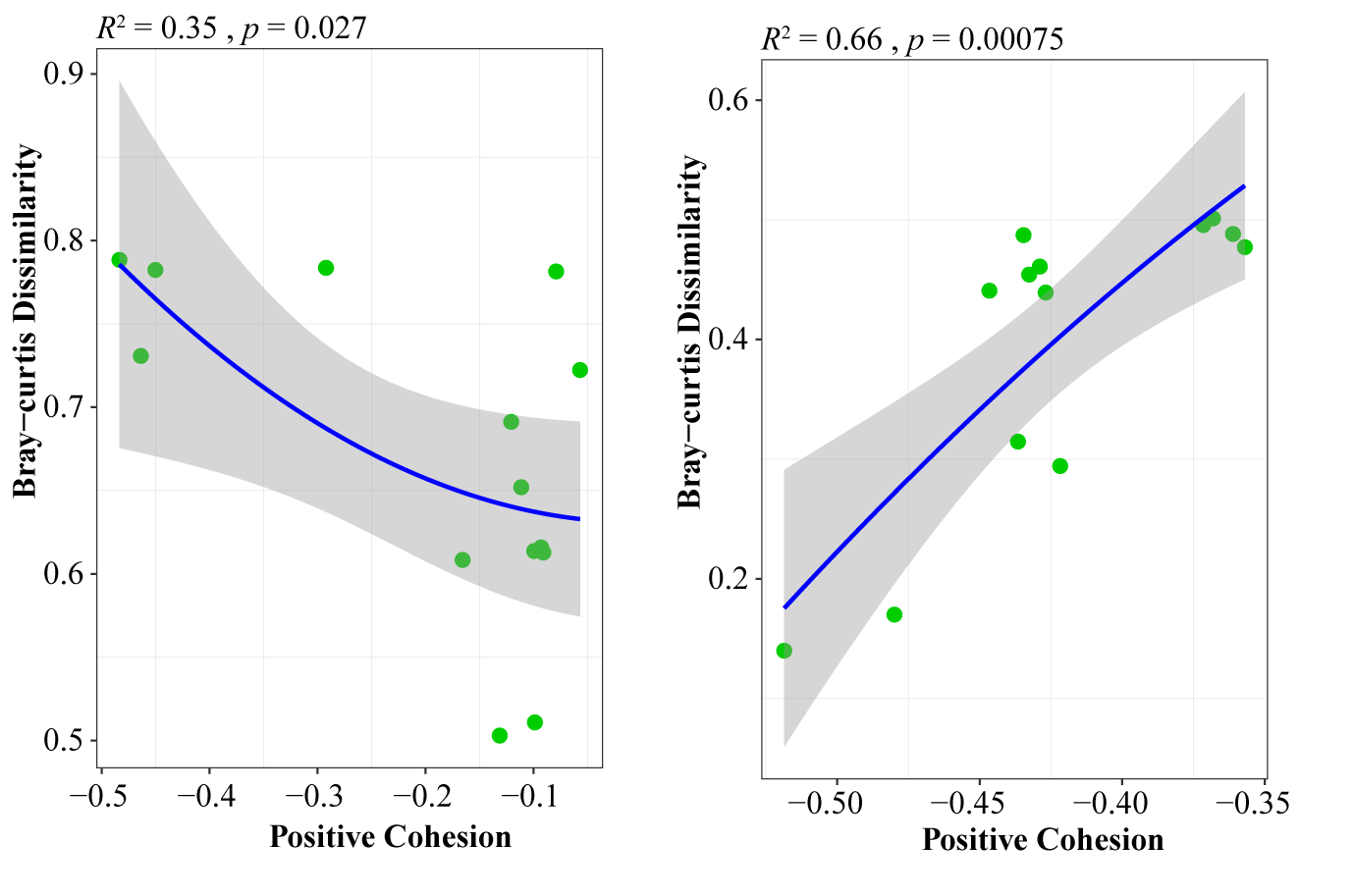

Supplement: Supplementary file 1 [file microorganisms-11-02498-s001.zip › FigureS2.tif]
